# Multicentre translational Trial of Remote Ischaemic Conditioning in Acute Ischaemic Stroke (TRICS): protocol of multicentre, parallel group, randomised, preclinical trial in female and male rat and mouse from the Italian Stroke Organization (ISO) Basic Science network

**DOI:** 10.1136/bmjos-2020-100063

**Published:** 2020-11-24

**Authors:** Mauro Tettamanti, Simone Beretta, Giuseppe Pignataro, Stefano Fumagalli, Carlo Perego, Luigi Sironi, Felicita Pedata, Diana Amantea, Marco Bacigaluppi, Antonio Vinciguerra, Alessia Valente, Susanna Diamanti, Jacopo Mariani, Martina Viganò, Francesco Santangelo, Chiara Paola Zoia, Virginia Rogriguez-Menendez, Laura Castiglioni, Joanna Rzemieniec, Ilaria Dettori, Irene Bulli, Elisabetta Coppi, Giorgia Serena Gullotta, Giacinto Bagetta, Gianvito Martino, Carlo Ferrarese, Maria Grazia De Simoni

**Affiliations:** 1 Department of Neuroscience Research, Istituto di Ricerche Farmacologiche Mario Negri Sede di Milano, Milano, Lombardia, Italy; 2 Department of Medicine and Surgery, University of Milan–Bicocca, Milano, Italy; 3 Department of Pharmacology, University of Naples Federico II, Napoli, Campania, Italy; 4 Department of Pharmacology, University of Milan, Milano, Lombardia, Italy; 5 Department of Pharmacology, University of Florence, Firenze, Toscana, Italy; 6 Department of Pharmacology, Università della Calabria, Arcavacata di Rende, Calabria, Italy; 7 Department of Neurology, San Raffaele Hospital, Milano, Lombardia, Italy

## Abstract

**Introduction:**

Multicentre preclinical randomised controlled trials (pRCT) are emerging as a necessary step to confirm efficacy and improve translation into the clinic. The aim of this project is to perform two multicentre pRCTs (one in rats and one in mice) to investigate the efficacy of remote ischaemic conditioning (RIC) in an experimental model of severe ischaemic stroke.

**Methods and analysis:**

Seven research laboratories within the Italian Stroke Organization (ISO) Basic Science network will participate in the study. Transient endovascular occlusion of the proximal right middle cerebral artery will be performed in two species (rats and mice) and in both sexes. Animals will be randomised to receive RIC by transient surgical occlusion of the right femoral artery, or sham surgery, after reperfusion. Blinded outcome assessment will be performed for dichotomised functional neuroscore (primary endpoint) and infarct volume (secondary endpoint) at 48 hours. A sample size of 80 animals per species will yield 82% power to detect a significant difference of 30% in the primary outcome in both pRCTs. Analyses will be performed in a blind status and according to an intention-to-treat paradigm. The results of this study will provide robust, translationally oriented, high-quality evidence on the efficacy of RIC in multiple species of rodents with large ischaemic stroke.

**Ethics and dissemination:**

This is approved by the Animal Welfare Regulatory Body of the University of Milano Bicocca, under project license from the Italian Ministry of Health. Trial results will be subject to publication according to the definition of the outcome presented in this protocol.

**Trial registration number:**

PCTE0000177.

## Introduction

Numerous treatments, which were reported to improve outcome in experimental animal stroke models, ultimately failed in clinical trials. Systematic reviews of experimental stroke research have consistently reported low-quality scores, negative publication bias and a paucity of data from female animals, aged animals or those with comorbidities, questioning the robustness and predictive value of single-laboratory preclinical experiments.^1^ To improve the translation of treatment efficacy from bench to bedside, the new concept of multicentre preclinical randomised controlled trial (pRCT) is emerging as a necessary step before moving from animal modelling to clinical trial.^2–4^ In multicentre pRCT, preclinical translational stroke research learns from the experience of clinical stroke research: a potential stroke therapy is tested under circumstances closer to the design and the rigour of a phase III randomised controlled clinical trial. A first rigorous pRCT on natalizumab in experimental ischaemic stroke has been successfully performed in Europe using two different models in mice.^5^ Notably, it showed remarkable similarity with the results of a concomitant multicentre clinical trial on the same drug in human ischaemic stroke.^6^

A nationwide network of preclinical stroke research laboratories, Italian Stroke Organization (ISO) Basic Science, has been created to perform multicentre translational research projects on highly promising therapeutic strategies in experimental ischaemic stroke, with the aim of overcoming the barrier between the bench and bedside. Remote ischaemic conditioning (RIC) represents a potential neuroprotective strategy in stroke. This procedure is aimed at triggering the activation of endogenous tolerance mechanism by delivering a subliminal ischaemic injury in the limbs, leading to a protective systemic response against ischaemic brain injury.^7 8^ Previous exploratory, single-centre studies have reported efficacy of RIC in reducing the consequences of ischaemic brain injury and have disclosed the pathophysiological mechanisms involved.^9^ This paradigm was chosen by the ISO Basic Science steering committee to enter a phase III multicentre pRCT to provide a strong statistical, analytical and reporting power, ultimately predicting drug efficacy before translation to clinic. RIC represents an ideal candidate to enter a multicentre pRCT with a rigorous study design,^10^ since previous results from single laboratories support its efficacy, but current phase II–III clinical trials provided inconclusive results.^11–14^ The project will investigate the efficacy of RIC in the experimental model of transient middle cerebral artery (MCA) occlusion in rats and mice of both sexes. Notably, ISO Basic Science network also obtained funding for a pilot clinical trial of RIC in patients with acute stroke who are not eligible for recanalisation therapies (intravenous recombinant tissue plasminogen activator and/or endovascular mechanical thrombectomy), which will be conducted in selected ISO-associated Stroke Units and will be part of a fully integrated translational research programme together with the preclinical trial.

## Study objective

This study aimed to test the hypothesis that hyperacute application of RIC increases the likelihood of good functional outcome, assessed using a dichotomised neuroscore at 48 hours, in transient MCA occlusion in male and female rats and mice. Secondary objectives are comparison of the means of the infarct volumes and comparison of the neuroscore as a continuous value between the two main experimental groups.

## Materials and methods

### Trial design

These are two multicentre, randomised, controlled, preclinical parallel trials, with an allocation ratio 10:10:2:2, blinded for outcome evaluation.

### Study setting

All experiments will be carried out in animal facilities of seven Italian academic or research institutions.

### Experimental groups

- MCA+/RIC+ (MCA occlusion, treated with RIC; treatment group) n=40 rats and n=40 mice.
- MCA+/RIC- (MCA occlusion, sham femoral artery surgery; control group), n=40 rats and n=40 mice.
- MCA-/RIC+ (sham carotid artery surgery, treated with RIC; single sham group), n=8 rats and n=8 mice.
- MCA-/RIC- (sham carotid artery surgery, sham femoral artery surgery; double sham group), n=8 rats and n=8 mice.

Details of experimental groups are shown in table 1.

**Table 1:** Experimental groups of the TRICS preclinical trial

Experimental groups	MCA+/RIC+ (Treatment)	MCA+/RIC- (Control)	MCA-/RIC+ (Single sham)	MCA-/RIC- (Double sham)
Total mice number (male/female ratio 1:1)	40	40	8	8
Mice per centre (four laboratories*)	10	10	2	2
Total rat number (male/female ratio 1:1)	40	40	8	8
Rats per centre (four laboratories*)	10	10	2	2

*Seven laboratories will participate in the project: three laboratories will use rats only, three laboratories will use mice only, one laboratory will use both rats and mice.

TRICS, Trial of Remote Ischaemic Conditioning in Acute Ischaemic Stroke.

### Choice of comparators

Control animals will receive a sham RIC since no acute treatment is currently recommended for acute ischaemic stroke after recanalisation therapies (intravenous rt-PA and/or endovascular mechanical thrombectomy). From a translational point of view, the animal stroke model employed in this study represents a large ischaemic stroke which achieves a futile reperfusion, that is, poor functional status despite successful reperfusion.

### Experimental models

1. Rats. Selected animals will be adult Sprague-Dawley rats, male and female 1:1 (regardless of oestrous cycle), weight 250 g±5%, housed in single cages, exposed to 12/12 hours light/dark cycle, at controlled room temperature, with free access to food and water, in specific pathogen-free facilities. Rats will be anaesthetised with 3% isoflurane in O_2_/N_2_O (1:3), maintained with 1.5% isoflurane in O_2_/N_2_O (1:3) and subjected to the occlusion of the origin of the right MCA for 100 min with a reperfusion period of 48 hours. Briefly, a silicone-coated filament (sized 5–0, diameter with coating 0.33 mm; coating length 5–6 mm; Doccol Corporation, Redlands, California, USA), will be introduced in the right external carotid artery and pushed through the internal carotid artery to occlude the origin of the right MCA. During MCA occlusion, rats will be awakened from anaesthesia, kept in a warm box and tested for the intra-ischaemic clinical assessment score in single cages. After 100 min, blood flow will be restored by carefully removing the filament, under anaesthesia. Treatment (RIC+ vs RIC-) will be applied after reperfusion (see below). During surgery, body temperature will be kept at 37°C by a heating pad. Sham rats will receive the same anaesthetic regimen and surgery than MCA occluded rats, their external and internal carotid arteries will be isolated but the filament will not be introduced. After surgery, all rats will be returned to single cages.
2. Mice. Selected animals will be adult C57BL/6J mice, male and female 1:1 (regardless of oestrous cycle), weight 26 g±5%, housed in single cages, exposed to 12/12 hours light/dark cycle, at controlled room temperature, with free access to food and water, in specific pathogen-free facilities. Mice will be anaesthetised by 3% isoflurane in O_2_/N_2_O (1:3) and maintained by 1%–1.5% isoflurane in O_2_/N_2_O (1:3). Transient occlusion of the origin of the right MCA will be induced for 60 min followed by a reperfusion period of 48 hours. A silicone-coated monofilament nylon suture (sized 7–0, diameter 0.06–0.09 mm, length 10±1 mm; diameter with coating 0.23 mm; coating length 2 mm, Doccol Corporation, Redlands, California, USA) will be introduced into the common carotid artery (appropriately isolated) and advanced to block the MCA. During MCA occlusion, mice will be awakened from anaesthesia, kept in a warm box and tested for the intra-ischaemic clinical assessment score in single cages. After 60 min, blood flow will be restored by carefully removing the filament, under anaesthesia. Treatment (RIC+ vs RIC-) will be applied after reperfusion (see "Interventions"). During surgery, body temperature will be kept at 37°C by a heating pad. Sham mice will receive the same anaesthetic regimen and surgery than MCA occluded mice, their external and internal carotid arteries will be isolated, but the filament will not be introduced. After surgery, all mice will be returned to single cages.

A maximum of four surgical procedures per day will be allowed.

Breath rate (number of breaths per minute) will be evaluated at baseline (before anaesthesia), after 15 min of anaesthesia and 15 min after withdrawal of anaesthesia, as a surrogate marker of anaesthesia tolerability.

Animals’ MCA+ without an ischaemic lesion at histology will be excluded a posteriori from any analysis, since they are not representative of an ischaemic individual (a human being would not have been randomised). Assessment of this exclusion criterion will be carried out by a researcher blinded to the randomisation arm and to the functional outcome.

The presence of two species with a different susceptibility to ischaemic injury (rat and mouse), as well as of the two sexes, aimed at reflecting a clinical patient cohort, strengthen the generalisability of the results.^15^

We expect that 25%–30% of animals will be excluded per the listed criteria.

### Interventions

RIC will be induced by surgical transient femoral artery occlusion. Briefly, at a predefined timing after reperfusion (20 min in rats and 10 min in mice), femoral artery will be identified, isolated and occluded with two microserrafine clips (Fine Science Tools) to stop the blood flow for a duration of 20 min in rats and 10 min in mice. The achievement of femoral artery blockade will be verified by visual inspection on the distal femoral artery territory. Sham-treated animals (RIC-) will receive the same surgery as that of RIC+ animals, their femoral arteries will be identified and isolated but not occluded.

### Health monitoring

Animals will be observed twice at 24 and 48 hours after surgery, before the behavioural testing. A predefined Middle Cerebral Artery Occlusion (MCAO) health report (available at https://figshare.com, DOI: 10.6084/m9.figshare.13031861), prepared on the basis of the Ischaemia Models: Procedural Refinements Of in Vivo Experiments (IMPROVE) guidelines,^16^ will be filled at baseline, at 24 hours and at 48 hours with information on animal welfare. Animals showing signs of moderate distress, according the MCAO health report, will receive subcutaneous buprenorphine 0.05–0.1 mg/kg every 8–12 hours (this dose is used for both rats and mice). Animals showing signs of severe distress, according to the MCAO health report, will be sacrificed before the end of the experiment. These animals, if sacrificed after RIC/sham application, will be retained in the intention-to-treat (ITT) analysis, and given the highest (ie, worst) score.

### Concomitant care

After surgery, animals will be housed in single cages. No concomitant treatment will be performed.

### Outcomes

#### Primary outcome

Difference in the proportion of rats (or, separately, mice) with a good functional outcome was measured by the dichotomised De Simoni composite neuroscore (13 items, range 0–56 points; available at https://figshare.com, DOI: 10.6084/m9.figshare.13031861)^4 17^ at 48 hours after MCA occlusion, that is, proportion of animals scoring 20 or less. Previous evidence from our group showed that the ischaemic lesion is fully developed histopathologically at 48 hours^12^ and the De Simoni neuroscore highly correlated with the histological assessment of infact volume (Pearson r=0.884, two-tailed p<0.0001, n=36, unpublished observations). A dichotomised functional outcome (0–20, good outcome; 21–56, poor outcome) was chosen according to the translational approach of this study. All clinical stroke trials uses dichotomised modified Rankin scale (0–2 vs 3–6) as the primary outcome measure,^18^ since this disability quantification is highly relevant for the patient. Inspired by clinical research, we decided to consider a cut-off of 20 for deficit dichotomisation using the De Simoni Neuroscore according to both animal behaviour and statistical issues. We indeed observed that MCA occluded mice scoring less than 20—at 48 hours after the ischaemic onset—have usually a small weight drop, index of preserved feeding and motility and increased chance to survive for long periods after experimental stroke. Moreover, when stratifying De Simoni neuroscores of MCA-occluded mice into quartiles, a score of 21 represented the 75 percentile.^19^ Unpublished results from our network suggest that the same cut-off is applicable also for MCA-occluded rats. In our view, a De Simoni neuroscore <20 corresponds, translationally, to a modified Rankin scale <2.

#### Secondary outcomes

1. Difference in infarct volume measured by volumetric histology at 48 hours after MCA occlusion.
2. Difference in the composite neuroscore (as a continuous variable) at 48 hours after MCA occlusion.

### Timeline

Experiments will be conducted between October 2020 and October 2021. The experimental plan will follow the timeline detailed in table 2.

**Table 2:** Timeline of the experimental plan

	Baseline (time 0)	24 hours after baseline	48 hours after baseline
Transient MCA occlusion/sham surgery	x		
Intraischaemic deficit evaluation	x		
Randomisation	x		
RIC±intervention	x		
Health report	x	x	x
Neuroscore			x
Sacrifice			x
Tissue collection			x
Vial sealing			x

MCA, middle cerebral artery; RIC, remote ischaemic conditioning.

### Sample size

Number of animals to be used in the experiments was defined separately by MCA arms. A formal procedure was used for the MCA+ groups, while a pragmatic approach was used for the MCA- groups. The same parameters were used for mice and rats. We used Power and Precision, V.4.1 (Biostat, Englewood, New Jersey, USA) for power calculations.

#### MCA+ arms

The primary objective of the proposed study is to test the null hypothesis that the proportion of good functional outcome (according to dichotomised De Simoni composite neuroscore) is identical in the two arms. From previous experiments (see "Primary Outcome"), we know that good functional outcome is present in approximately 20% of the RIC- animal. We posed that the minimum difference in proportions should be equal to or higher than 30 percentage points to be of substantive translational significance, that is, RIC+ animals should have a good functional outcome of 50% against the standard 20% in RIC- animals. These figures are equivalent to a relative risk of 2.5 of a good outcome (50% vs 20%) and a relative risk of 0.625 of a bad outcome (50% vs 80%) of the treatment against no treatment. The criterion for significance (alpha) was set at 0.050, two-tailed. With a sample size of 40 for each of the two groups, the study will have a power of 82% to yield a statistically significant result, using a χ^2^ test as a proxy for a more complex logistic regression model involving sex and centres. Each of the two trials will therefore have a total of 80 animals randomised to the MCA+ arms.

#### MCA- arms

No specific statistical analysis was carried out to determine the number of animals allocated to the two MCA- arms, since they will only serve as internal controls for neurobehavioural assessment: we already know that the mean neuroscore values for MCA- will be 2.2 (0–6 min and max values, as per blinded assessments reported previously^20^ and unpublished data), thus being lower than the best score in the MCA+ group. We however anticipate that four animals per centre per MCA- group will be randomised, keeping their number at a minimum, in line with the ethical requirement of reducing the number of animals used for preclinical research. The sham animals will serve as baseline for the outcome measures expressed as fold-change from baseline and to descriptively assess possible harms due to RIC.

### Correction for loss of animals due to violations of inclusion and exclusion criteria

Considering that approximately 30% of the animals will not be available for the analysis (according to the inclusion and exclusion criteria), we anticipate that we will need 120 total animals per species.

### Inclusion and exclusion criteria

Rats and mice will be included in the study if cerebral ischaemia is successfully induced, that is, filament correctly positioned in the MCA origin. After MCA occlusion, the following intraischaemic clinical assessment score will be applied. Animals will be judged ischaemic, and included in the trial, if presenting ≥3 of the following deficits after filament insertion:

1. The palpebral fissure has an ellipsoidal shape (not the normal circular one).
2. One or both ears extend laterally.
3. Asymmetric body bending on the ischaemic side.
4. Limbs extend laterally and do not align to the body.

Animals will be excluded in case of:

1. Death during MCA surgery or in any case before RIC procedure.
2. Major experimental protocol violations: errors or surgical complications (eg, major arterial or venous haemorrhage, section of the vagus nerve, carotid artery dissection, filament entrapment or displacement) during MCA occlusion procedure; errors in ischaemia time; errors or surgical complications (eg, major arterial or venous haemorrhage, section of the sciatic nerve, clip misplacement) during RIC procedure; errors in RIC timing.

### Randomisation

#### Sequence generation

Two randomisation lists will be produced, separately by species and stratified by centres and sex. The lists will be produced using a pseudo-random number generator, using permuted blocks of random size: a procedure implemented using JMP Pro will be used to generate the list. Random numbers inside JMP Pro are generated using the Mersenne-Twister technique. Every centre will randomise according to a given list containing all four groups, stratified by species and sex (MCA+ and MCA-, RIC+ and RIC-). Allocation ratio (MCA+/RIC+: MCA+/RIC-:MCA-/RIC+:MCA-/RIC-) will be (10:10:2:2). Since the two sexes will be equally represented, for each sex every centre will treat 5+5+1+1 animals, with three available replacements.

#### Animal replacement

Animals sacrificed before RIC application will be replaced, up to a total number of three per each centre, per species and per sex. We can anticipate that replacement will be done almost exclusively on the animals receiving the MCA+ treatment. In any case, the new animal will receive the same MCA treatment of the replaced animal. Formally, this means that new animal will not be subject to a randomisation to MCA+/MCA. However, this *missing randomisation* does not have an implication on the primary analysis, since this analysis will be done only on the difference between RIC+ and RIC-, and this last randomisation takes place independently and after MCA intervention.

### Concealment

Each centre will receive 30 sealed, non-transparent, non-resealable envelopes per treated species. Envelopes will be marked with a code from Male01 to Male15 and from Female01 to Female15, and a sketched mouse or rat, with, respectively, an M or an R letter. Envelopes were preferred to online randomisation due to logistic constraints (unavailability of online access in animal care zones). Centres can treat mice, rats or both. In case they treat the two species, they will receive 60 envelopes. After envelope opening, the sheet containing centre identification, male/female, progressive number and pretreatment (MCA+/MCA-) assignment, will be signed and dated. The external envelope will contain another envelope: this internal envelope, containing the treatment option (RIC+/RIC-) will be opened after surgery for MCA occlusion in order to guarantee allocation concealment to surgeons. Sheet containing RIC treatment will also be signed and dated. Sealed envelopes will be stored in different, locked, places from signed and dated sheets.

### Implementation

Randomisation lists and envelopes containing randomisation treatment allocations will be prepared by personnel not involved in the implementation of procedures with the animals. The two original lists will be stored as encrypted files in a secure, backed up server and will also be printed and stored in a locked cabinet. The specific details of the procedure used to randomise will be briefly described in a document stored together with the definitive lists.

### Blinding

Inclusion criteria will guarantee blinding, since the intraischaemic clinical assessment score is applied before randomisation. Exclusion criteria will be applied after surgery in a blinded manner by the same researchers assessing functional outcome. Assessment of functional outcome measured by the De Simoni neuroscore will be conducted locally by researchers blinded to MCA occlusion (MCA+ vs MCA-), treatment allocation (RIC+ vs RIC-) and cause of any death. No person assisting the surgeon in any way will carry out assessment of functional outcomes. Histological outcome will be centralised to the coordinating centre (University of Milano Bicocca) and performed by a single experienced researcher blinded to group allocation. Data analysis (RIC+ vs RIC-) will be conducted blinded to the group allocation. Unblinding will be performed by the statistician after the final results of statistical analysis will be reached.

### Data collection methods

#### Primary outcome

A centralised training for a correct administration of the De Simoni composite neuroscore will be performed by the coordinating unit in multiple meetings before starting animal randomisation and providing a video to show in a detailed way the execution of the assessment. In order to limit excessive variability in the interpretation of the De Simoni’s neuroscore, we planned to provide a video tutorial to each centre to explain how to carry out the test. Then the operators involved in the behavioural assessment will be tested by evaluating—blinded to the experimental conditions—different videos. The assigned score will be analysed centrally to provide a feedback to limit subjective evaluations by the operators and calculate a Fleiss kappa coefficient for inter-rater agreement. The video tutorial and operator testing will be coordinated by the De Simoni’s group. The protocol for composite neuroscore has been previously published.^4^ In adjunct to the training in performing the behavioural evaluation, the personnel will be given specific instructions to collect and input the relative data.

#### Secondary outcomes

- Infarct volume. Whole intact brains will be fixed in 10% neutral buffer formalin for 24–48 hours at 4°C, then transferred in phosphate buffered saline +0.05% sodium azide at 4°C and sent to the coordinating unit. Coronal sections (50 µm) will be stained using cresyl violet 0.1%. Infarct areas will be measured in consecutive sections with 250 µm interval. Infarct volume will be calculated using ImageJ image processing software (National Institute of Health, Bethesda, Maryland, USA) and expressed in mm^3^. Infarct volume will be corrected for cerebral oedema with the following equation: (ischaemic area) = (direct lesion volume) − ((ipsilateral hemisphere) − (contralateral hemisphere)). Infarct volume will be measured centrally by the coordinator unit.
- Original composite neuroscore (as a continuous variable) at 48 hours after MCA occlusion will also be retained.

### Data management

Study data will be collected and managed using REDCap^21 22^ electronic data capture tools hosted at the Istituto di Ricerche Farmacologiche Mario Negri IRCCS on behalf of the coordinating unit. REDCap (Research Electronic Data Capture) is a secure, web-based software platform designed to support data capture for research studies, providing (1) an intuitive interface for validated data capture; (2) audit trails for tracking data manipulation and export procedures; (3) automated export procedures for seamless data downloads to common statistical packages; and (4) procedures for data integration and interoperability with external sources. REDCap is at present working on a Apple XServe web server (Quad-core Intel Xeon at 2.26 GHz and 12 Gb RAM) with OS X V.10.11 ‘El Capitan’, and a MySQL server mounted on an HP DL380 Gen10 (2 Intel Xeon 4110 8-Core/2.1 GHz and 32 Gb RAM) with SLES SuSE V.12 as operating system. Data will be backed up daily.

Health reports and composite neuroscores will be recorded in a specific laboratory notebook with preformatted forms, containing spaces for dates and signatures of the persons in charge of the single experiment. Within 48 hours after their collection, data will be uploaded in the web-based software platform, with automatic controls ensuring that complete information is provided (refer to the health report spreadsheet and to the neuroscore protocol). Mandatory fields will not be used to block inputing. Automatic reports pointing at missingness and possible inconsistencies will always be available, separately for every animal, and will also be sent regularly via email to the centres.

In every centre, a specific person (PRI: person responsible for inputing) will be provided with a personal username and a password that will be used to input data.

### Statistical methods

#### Outcomes

Twin trials (mouse and rat) data will be analysed separately, with identical statistical methods, and statistical significances will be assessed separately. The following cases can be obtained:

- Both twin trials give a (positively) statistically significant result: the overall result is considered positive.
- Both twin trials give a statistically non-significant result: the overall result is considered negative.
- A trial results in a (positively) statistically significant result and the other trial results in a statistically non-significant result: since the combination of the results can lead to a lower overall power than that specified for the single trials, at the prespecified 0.05 alpha level, the raw data from the two trials will be combined and a test will be conducted: the overall result will be obtained from this procedure.

In any case, results from the single trials will be reported and tests on the combination will be carried out only in case of discordance.

Comparisons will be made only between MCA+/RIC+ and MCA+/RIC- groups, while MCA-/RIC- and MCA-/RIC+ groups will only be analysed from a descriptive point of view. Note: from here on, only MCA+ animals are the subject of the analyses.

Cut-off for statistical significance will be set at 0.05, two-tailed. Analyses will be performed in a blinded status. Analyses will be performed using Stata/IC V.15 or higher (Statacorp). Data preprocessing and descriptive analyses will be performed using JMP Pro V.14 or higher (SAS Institute).

We report below specific analyses for the main variables.

#### Baseline description of the groups

Descriptive analyses will be carried out using classification (number and percentages) in categorical variables (ie, sex) and using graphical methods (histograms) and means, SD, medians and quartiles in numerical variables (number of animals per cage, body weight).

#### Primary analysis

The proportion of animals with good neurological outcome at 48 hours after the intervention will be compared between RIC+ and RIC- groups by means of a logistic regression. The only covariate included in the model will be sex, using centre as a random effect. Two models will be compared, and one will be selected as the primary one. The first model will only include main effects, while a second model will also include the interaction of RIC by sex (irrespective of the significance of the main effects). Log likelihood of the two models will be compared and the second model will be selected as the primary one if the likelihood ratio test between the two gives a p value lower than the predefined cut-off for statistical significance.

#### Secondary analyses

The principal secondary analyses will be performed looking at the infarct volume, expressed as a continuous variable (mm^3^). The effect of the treatment will be studied using a mixed linear regression model, with sex as the only covariate, using centre as a random effect. Interaction between sex and treatment will also be tested in a separate model. Heteroscedasticity will be checked graphically on the residuals of the single models: in case of non-constant variance, a robust estimator will be used. A jackknife method will be used to assess presence of leverage points.

Another analysis will be performed using the original neuroscore value (not dichotomised) as an outcome, using a mixed linear regression, in a model which contains sex, and centre as a random effect. Presence of leverage points and of heteroscedasticity will also be assessed.

Female animals were included in the experiment to take into account the presence of both genders among the human patients. Even though the experiment was not powered to answer the questions if a difference exists between sexes in response to the treatment, we think that using both sexes is an important step towards translational medicine, and that even preliminary data can be of value.

Descriptive analyses with means, medians, SD and quartiles, and graphical methods will be carried out on the raw values of the neuroscore by treatment (and also by treatment by sex) in order to inspect the effect of the treatment in (possibly) reducing the percentage of animals with bad functional outcome.

Health report classes will be descriptively reported at all available time (0, 24 and 48 hours) presenting numbers and percentages.

#### Subgroup/adjusted analyses

The same primary and secondary outcomes will be reanalysed in the subgroup of animals reaching alive the 48 hours after MCA occlusion.

#### Population analysis and missing data

Primary analyses will be conducted according to an ITT paradigm, including all RIC+ randomised animals (with the exception of animals without sign of ischaemia at neuropathological inspection, since these cannot be considered a model of ischaemic stroke). We do not anticipate presence of missing data: should some data on the neuroscore be lost, presumably due to procedural problems, we will adopt a multiple imputation model with 50 imputations. In case an animal dies (after RIC) before being evaluated at 48 hours, it will be given the worst attainable score (and therefore it will be counted as a negative outcome).

### Data monitoring: formal committee and interim analysis

Due to the short time involved in the execution of the experiments and the absence of recruitment problems, no data monitoring committee will be appointed.

Since the proportion of animals unavailable for the analysis (due to premature death or absence of ischaemia at neuropathological examination) is highly variable, the steering committee will perform a recruitment control at each centre when approximately 50% of the experiments have been performed, checking that the percentage of excluded animals is not superior to the estimate (30%). A higher value will require reassessment of quality standards for these centres and may imply exclusion from the study.

An interim analysis of primary outcome and secondary outcomes is not planned.

### Harms

The available time to record adverse effects of treatment is limited, therefore no long-term or chronic outcomes can be recorded.

Nevertheless, we will assess the presence of the following, at 24 and 48 hours:

- Gait abnormalities.
- Local signs of inflammation/infection in the hindlimb treated with RIC.

Since these symptoms will be in all probability hidden by the presence of the MCA+ intervention, they will be closely controlled in MCA- animals. However, due to the low number of MCA- animals, only a descriptive analysis will be provided.

### Auditing

Quality assessments will be carried out by the coordinating unit using on-site visits and remote form checking. A manual check will be performed by a researcher from the coordinating unit in charge of monitoring (CUCP: coordinating unit checking person). The CUCP will have access in read mode to all records, while the PRIs will be the only with the permissions to input/modify data in the database, for their specific centre.

The CUCP will also be in charge of visiting the centres to check the consistent application of the procedures and to try to resolve pending problems. The CUCP will also be present in person in each local centre at the time of execution of the first experiment in order to verify the correct application of the trial rules. A written report will be compiled for each visit. A final visit to each centre will be carried out after the end of the experiments to clear the pending queries and collect as much as possible data. On-site visits will check inclusion and exclusion criteria, correspondence between handwritten and web form inputed data, presence of violations of protocol, presence of sacrificed animals before the end of the study, envelopes conditions and participation to the centrally coordinated meetings. A specific data monitoring plan will be created before the beginning of the study.

No external auditing is planned.

### Protocol amendments

In case of presence of protocol amendments, all of them will be recorded on a specific document that will be integral part, as an appendix, of the final report.

Any amendment will have to be approved by the steering committee, and, if substantial, notified to the Ministry of Health. Substantial amendments will require a change in the protocol version number.

### Access to data

During the course of the trial, the database will be curated by a subunit based at the Istituto di Ricerche Farmacologiche Mario Negri IRCCS, and access will be available only for input and verification.

On completion of the trial, a complete cleaned dataset will be prepared, together with its related dictionaries. This dataset will be made available on the publication of the scientific papers using online free data repository like Figshare, under the CC BY 4.0 license.

### Ancillary and post-trial care

Not applicable.

### Pre-registration

Registered at https://preclinicaltrials.eu, identifier PCTE0000177, published on 6 January 2020. Log in required to access records.

### Dissemination policy

#### Trial results

Trial results will be subjected to publication in a peer-reviewed journal irrespective of the final results of the study. Publication will be based on the definition of the outcome presented in this protocol. Successive publications could also be written on specific aspects of the experiments. The steering committee will be responsible for the approval of the papers.

#### Reproducible research

Raw data will be available on the publication of the scientific papers using online free data repository like Figshare, under the CC BY 4.0 license. The link to the data repository will be available from the published paper and the preclinical trial registry preclinicatrials.eu.

#### Biological specimens

Not applicable.

#### Sponsor

University of Milano Bicocca, Piazza dell’Ateneo Nuovo 1, 20 126 Milano, Italy.

#### Steering committee

The steering committee will be composed by the main authors of this preregistration report and headed by Professor Carlo Ferrarese, principal investigator of the study.

**Strengths and limitations of the study**

- Strengths of the study include the following: RIC represents an ideal candidate to enter a multicentre pRCT, since previous results from single laboratories support its efficacy, but current phase II–III clinical trials provided inconclusive results; a rigorous study design according to the Landis 4 criteria; multiple species assessed; both sexes included.
- Limitations of the study include a severe ischaemic stroke model of large vessel occlusion, which limits generalisability to other ischaemic stroke subtypes (lacunar strokes, minor strokes); a short-term outcome of 48 hours, which limits the results to the acute phase and does not investigate the long-term recovery phase.
- The TRICS (Trial of Remote Ischaemic Conditioning in Acute Ischaemic Stroke) preclinical trial will provide robust, translationally oriented, high-quality evidence on the efficacy of RIC in multiple species of rodents with large ischaemic stroke. The results of this study may promote translation of RIC into further randomised clinical trials.
